# Protocol for paired isolation and characterization of migrating cancer cell subpopulations in 3D hypoxic microenvironments

**DOI:** 10.1016/j.xpro.2026.104847

**Published:** 2026-09-19

**Authors:** Luana Schito, Sergio Rey-Keim

**Affiliations:** 1 UCD School of Medicine and UCD Conway Institute of Biomolecular and Biomedical Research, University College Dublin, Belfield, D04 C7X2 Dublin, Ireland

**Keywords:** Cell Biology, Cancer, Molecular Biology, Organoids

## Abstract

Paired isolation and characterization in 3D hypoxic microenvironments (HyPIC-3D) is a platform for delineating migratory heterogeneity among cancer cell subpopulations within 3D hypoxic tumor microenvironments. Here, we present the protocol for implementing HyPIC-3D using standard laboratory and computational resources. We describe the steps to perform migration assays with cancer spheroids, collect paired non-migrated and migrated subpopulations, and characterize their heterogeneous phenotypes through quantitative imaging, molecular, and cellular analyses. This protocol has potential for applications in diverse 3D migration contexts.

For complete details on the use and execution of this protocol, please refer to Schito and Rey-Keim^1^

## Highlights

• Instructions for carrying out migration assays within cancer spheroids
• Steps for isolating and collecting migrating and non-migrating cancer cells
• Transcriptional and hypoxic profiling of migrating and non-migrating cancer cells
• Quantitative analysis of migratory phenotypes

## Abstract (editor highlights)

Publisher’s note: Undertaking any experimental protocol requires adherence to local institutional guidelines for laboratory safety and ethics.

## Graphical abstract

**Figure:**
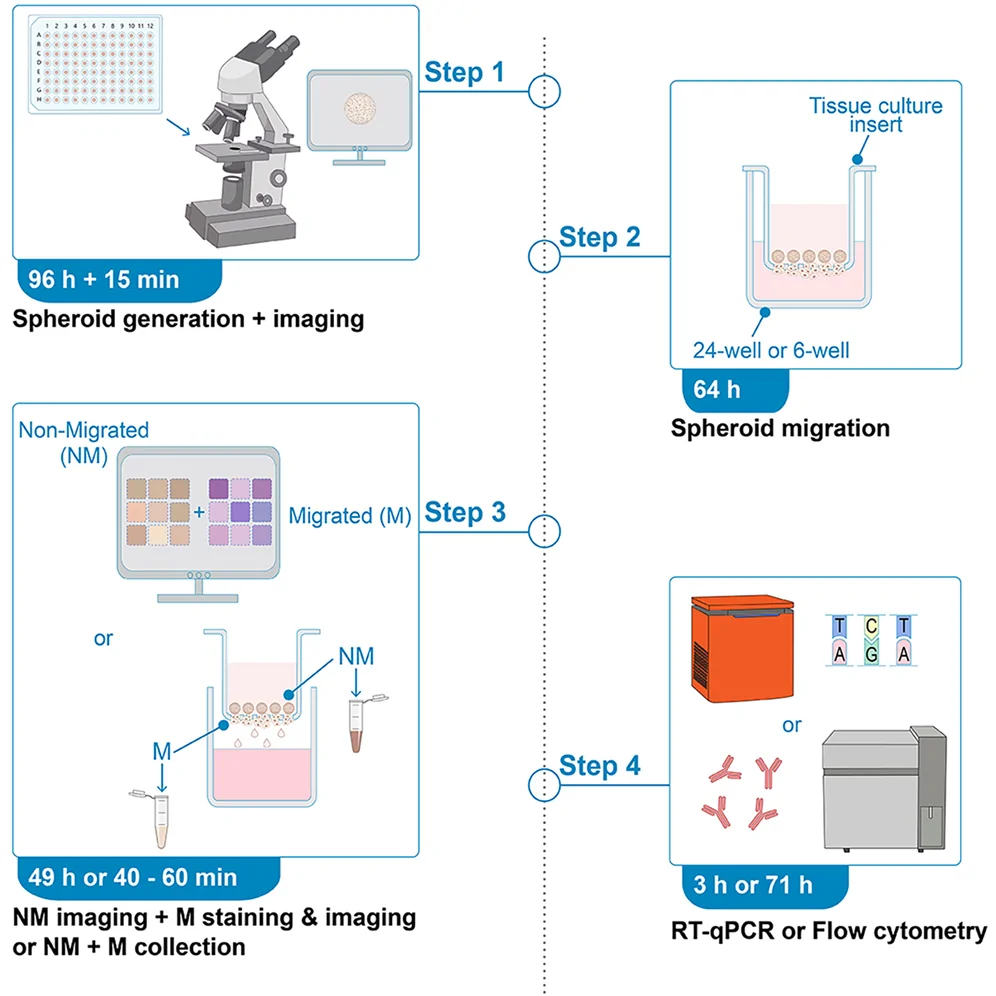

## Before you begin

Human cancers contain heterogeneous migratory and non-migratory cell populations influencing the metastatic process. Delineating the mechanisms promoting this functional heterogeneity is a major challenge in cancer research that can be tackled by an experimental setup enabling side-by-side dissection of these populations. The HyPIC-3D protocol is designed to allow isolation of migrating and non-migrating cancer cell subpopulations from a common spheroid pool. Collected samples are then amenable to downstream molecular and cellular analyses, generating datasets that can be interrogated by paired statistical testing; this approach improves sensitivity and allows to correlate migratory behaviors with distinct molecular and cellular phenotypes.

HyPIC-3D is optimized with 3D cultures that recapitulate key features of the *in vivo* tumor microenvironment, including spatial heterogeneity, O_2_ gradients and ultimately, hypoxia, a well-established driver of cancer cell migration and metastasis,^2^^,^^3^ further increasing the pathobiological relevance of the findings. Moreover, hypoxia in HyPIC-3D is not imposed by default through external sources such as hypoxic workstations or chambers, but arises intrinsically within the spheroid microenvironment through O_2_ diffusion limits and cell-autonomous O_2_ consumption, generating spatial O_2_ gradients driving migratory heterogeneity.

Building on this background, the HyPIC-3D protocol describes procedures for spheroid generation and migration, outlining steps for sample collection and processing at migration endpoint. In this setup, migration reflects the active movement of cells as they remodel the spheroidal matrix within their 3D environment, migrate through it, and eventually reach the insert membrane, a physical barrier that cells must actively cross. HyPIC-3D also provides steps for quantitative morphometric analysis of migratory phenotypes using automated and semi-automated codes available with the protocol. Included are also procedures for applying HyPIC-3D to downstream molecular and cellular applications such as paired transcriptional profiling and intracellular hypoxia assessment in migrated and non-migrated fractions by reverse transcription quantitative PCR (RT-qPCR) and flow cytometry, respectively. The HyPIC-3D protocol has been successfully applied across a range of cancer cell types to delineate differences in migratory behavior, gene expression, and intracellular hypoxia between migrating and non-migrating subpopulations.^1^

Before starting the protocol, users should:

1. Refer to the literature regarding the ability of the cancer cell line under investigation to form spheroids (e.g., ref.^4^), followed by validation under user’s specific experimental conditions.
2. Consider that HyPIC-3D is validated with spheroids generated without the addition of exogenous matrices. Accordingly, the experimental procedures outlined within this protocol are implemented with the lung cancer cell line A549, which forms spheroids of good compaction.

***Note:*** HyPIC-3D has been also validated in HCT116, HeLa and VHL-null RCC4 cancer cell lines. These lines were maintained under standard culture conditions (i.e., RPMI-1640 + GlutaMAX or DMEM high glucose, 10% FBS, and supplements according to cell line; incubated at 19% O_2_, 5% CO_2_ and balance N_2_ at 37°C) and formed spheroids under the same experimental conditions used for A549 cells described herein, without added matrices.^1^

***Note:*** Environmental O_2_ in a standard incubator varies with altitude and incubator setup.

3. Optimize microscopy setup for uniform illumination.
  a. Use a microscope equipped with a stable LED illumination source.
  b. Ensure even illumination and contrast across the field of view prior to imaging.
  c. Apply flat field correction if necessary.

### Innovation

The HyPIC-3D protocol addresses limitations of current 2D and 3D migration (and invasion) systems in performing side-by-side (i.e., paired) analyses of migrated and non-migrated cancer cell subpopulations at the assay endpoint, an aspect critical for characterizing migratory heterogeneity within a clonal population. Accordingly, one key innovation of this protocol is the simultaneous recovery of both subpopulations from a common batch of spheroids for downstream, paired analyses. This approach also eliminates the need for post-assay cell culturing to increase sample yield, enabling a more direct correlation between molecular profiling and the observed migratory phenotypes. The HyPIC-3D protocol also provides semi-automated imaging pipelines that extract morphometric parameters such as extent, density and spatial pattern of migration, reducing operator bias and improving reproducibility. HyPIC-3D uses standard tissue culture (TC) inserts commonly employed in Boyden chamber assays, so that the protocol can be executed with standard laboratory equipment. Finally, by using spheroids, HyPIC-3D interrogates migration in 3D *in vitro* microenvironments that retain cell-cell and cell-matrix interactions and intrinsic hypoxia, more closely reflecting *in vivo* malignant contexts.

### Cell thawing and culture

**Timing: 1 week**

Thaw A549 cells at least one week ahead of spheroid generation to allow their full recovery from cryopreservation.

***Note:*** Timing may vary depending on cell viability and confluency at the moment of thawing.

4. Quickly thaw the cryovial containing A549 cells in a 37°C water bath.
  a. Bring the cryovial to a laminar flow hood and gently pipette the cells.
  b. Transfer cells to a 75 cm^2^ flask containing 10 mL of pre-warmed, complete growth medium.
    ***Alternatives:*** Transfer cells from the cryovial to a 15 mL conical tube containing fresh medium and centrifuge (e.g., 200–300 × *g* for 5 min at 20°C–25°C) to remove the cryoprotectant dimethyl sulfoxide (DMSO). Cells can now be seeded into the flask.
  c. Inspect cells under the microscope.
    ***Note:*** In this step users can check cell viability and cell number via Trypan blue exclusion assay.
5. Place A549 cells in a humidified incubator (>90% relative humidity) at 19% O_2_, 5% CO_2_ and balance N_2_ at 37°C.
6. Replace growth medium with fresh medium after 24 h. Check cell confluency under a microscope.
7. Return A549 cells to the incubator and maintain them by regularly replacing growth medium and by passaging them according to confluency and doubling time.

***Note:*** We typically passage A549 cells when they reach ≈ 70% confluency in order to avoid over-confluency that would forestall exponential growth.

**CRITICAL:** Cells should be routinely screened for Mycoplasma contamination; MycoStrip can be used for this testing.

## Key resources table

**Table:** 

REAGENT or RESOURCE	SOURCE	IDENTIFIER
**Antibodies**		

Hypoxyprobe™ Mab-1 Antibody, used as 1:250 dilution	Hypoxyprobe	Cat# HP MAb-1; RRID: AB_2801307
Goat Anti-Mouse IgG (H+L) Antibody, Alexa Fluor-647 Conjugated, used as 1:1000 dilution	Molecular Probes	Cat# A-21235; RRID: AB_2535804

**Chemicals, peptides, and recombinant proteins**		

Crystal violet	Merck	Cat# C6158-50G
Paraformaldehyde Solution, 4% in PBS	Thermo Scientific	Cat# J19943.K2
RPMI-1640 + GlutaMAX	Gibco	Cat# 61870036
FBS	Gibco	Cat# A5670701
Penicillin/streptomycin	Gibco	Cat# 15140122
Trypsin-EDTA (0.05%)	Gibco	Cat# 25300054
Trypan Blue solution, 0.4%	Gibco	Cat# 15250061
Triton X-100	Fisher BioReagents	Cat# 10102913
Methanol	Fisher Chemical	Cat# 10675112
Bovine Serum Albumin (BSA)	Merck	Cat# A7030-10G

**Critical commercial assays**		

MycoStrip	InvivoGen	Cat# rep-mysnc-50
RNeasy Mini Kit	QIAGEN	Cat# 74106
FIREScript RT cDNA synthesis MIX	Solis BioDyne	Cat# 06-20-00100
Hot FIREPol EvaGreen qPCR Mix Plus	Solis BioDyne	Cat# 08-25-00001–10
Hypoxyprobe Kit (for mouse MAb)	Hypoxyprobe	Cat# HP1-100Kit

**Experimental models: Cell lines**		

A549 cells	Merck	Cat# 86012804-1VL

**Software and algorithms**		

CytExpert	Beckman Coulter	RRID:SCR_017217
FlowJo v10.10	BD BioSciences	RRID:SCR_008520
GraphPad Prism v10.6.1	GraphPad Software	RRID:SCR_002798
Fiji v1.54p	ImageJ	RRID:SCR_002285
Q-qPCR v1.0.4	Bio Molecular Systems	N/A
MII ImageView software v4.12	BestScope	N/A
Script 00 Automated conversion of phase-contrast images to 8-bit grayscale	Schito & Rey-Keim^1^	https://github.com/LS2RLab/HyPIC-3D or https://doi.org/10.6084/m9.figshare.30010504
Script 01 Automated HyPIC-3D algorithm for spheroid image analysis at growth endpoint	Schito & Rey-Keim^1^	https://github.com/LS2RLab/HyPIC-3D or https://doi.org/10.6084/m9.figshare.30165115
Script 02 Semi-automated HyPIC-3D algorithm for image analysis of spheroids at migration endpoint	Schito & Rey-Keim^1^	https://github.com/LS2RLab/HyPIC-3D or https://doi.org/10.6084/m9.figshare.30165133
Script 03 Semi-automated HyPIC-3D algorithm for image analysis of post-migrated, crystal violet-stained spheroids	Schito & Rey-Keim^1^	https://github.com/LS2RLab/HyPIC-3D or https://doi.org/10.6084/m9.figshare.30165136

**Other**		

Ultra-low attachment (ULA) 96-well microplates	Corning	Cat# 7007
Tissue culture inserts (24-well plates; pore size: 8 μm)	Sarstedt	Cat# 83.3932.800
Tissue culture inserts (6-well plates; pore size: 8 μm)	Sarstedt	Cat# 83.3930.800
Countess 3	Thermo Fisher	Cat# AMQAX2000
Countess Cell Counting Chamber Slides	Thermo Fisher	Cat# C10228
Swabs	Texwipe	Cat# TX759B
Cotton swabs	Texwipe	Cat# STX705W
Microscope CKX53	Olympus	SKU: CKX53
Low-retention pipette tips	Fisher Scientific	Cat#: 10571963
Pipetman 8-channel	Gilson	Cat# FA10011
Pipetman12-channel	Gilson	Cat# FA10012
Reagent reservoirs	Merck	Cat# BR703459-10EA
Centrifuge 5810 R	Eppendorf	Cat# 5811000061
Swing-bucket rotor A-4-62	Eppendorf	Cat# 5810709008
Microtiter plate bucket	Eppendorf	Cat# 5810702003
Q-qPCR Instrument	Quantabio	Cat# 95900-4C
Thermal cycler, Ristretto (32-wells)	VWR	Cat# 732–2553
Nanodrop 2000 spectrophotometer	ThermoFisher Scientific	Cat# ND-2000; RRID:SCR_018042
CytoFLEX S V2-B2-Y3-R2	Beckman Coulter	Cat# B96621
Roto-mini plus rotator	Gilson	Cat# 36110120

## Materials and equipment

### Ultra-low attachment 96-well microplates for spheroid generation

We use ULA microplates as preferred method to generate spheroids. Per each microplate, we generate 96 individually grown spheroids of consistent size, roundness and compactness.

***Alternatives:*** In-house made, agarose-coated, U-bottom 96-well microplates. Various agarose-coating protocols are available in the literature; see for example Friedrich J, et al.^5^

### Multi-channel pipettes and reagent reservoirs

We use either an 8- or 12-channel pipette to minimize cell sedimentation during seeding and to ensure spheroid size consistency within and among independent experiments. Along the same lines, we use autoclavable polypropylene reagent reservoirs with a robust construction and a V-shaped bottom to prevent movement during pipette loading and to minimize dead volume, respectively.

### Centrifuge

We use a centrifuge with a swing-bucket rotor and a microplate adapter to spin ULA microplates immediately after seeding A549 cells. This step brings cells to the center of each well, forming an initial uniform 2D disc, accelerating aggregation and subsequent formation of uniform spheroids.

### Standard tissue culture incubator

A dedicated tissue culture incubator for spheroid growth, migration assays and pimonidazole staining is preferable as it allows the spheroids to remain undisturbed throughout these procedures.

### TC inserts

We use TC inserts with polyethylene terephthalate (PET) membranes in both 24-well and 6-well formats with pores of 8 μm diameter (see key resources table), allowing migration of A549 cancer cells while precluding passive fall-through; this can be adjusted according to the size of the migrating cell type, employing pores of different sizes (e.g., 5.0 μm or 3.0 μm) where required, for example when adapting this protocol to 3D models other than homotypic A549 spheroids, such as heterotypic cancer spheroids, patient-derived organoids, circulating tumor cell clusters and mammospheres,^1^ provided cohesion is sufficient to withstand transfer between culture vessels (see limitations). In addition, we use uncoated PET membranes to measure migration. Exogenous matrix coating, for example with Matrigel, can be employed where invasion is the intended readout.^1^

### Microscope equipped with a 2× objective

A 2× objective allows to acquire four consecutive images covering the whole PET membrane; these images are then stitched and analyzed.

### Image acquisition software

Image acquisition throughout this protocol is performed using MII ImageView software v4.12.

### Swabs

Swabs made of 100% polyester, nonwoven material are essential to the efficient detachment and recovery of the non-migrated spheroid fraction from the TC insert at migration endpoint, acting as lifters rather than as cleaning swabs.

**CRITICAL:** Nonwoven swabs should be used at lifting steps, as spheroids might become embedded/trapped within the cotton fibers. This is especially critical when the non-migrated fraction is recovered for downstream analysis, to avoid sample loss. Cotton swabs remain suitable for washing and cleaning the inserts.

***Alternatives:*** Mini polyethylene cell scrapers fitting within the dimensions of 24-well and 6-well TC inserts.

**Table:** Complete cell and spheroid growth medium

Reagent	Final concentration	Amount
RPMI-1640 + GlutaMAX	N/A	445 mL
FBS	10%	50 mL
Penicillin/streptomycin	1%	5 mL
Total	–	500 mL

***Note:*** Store at 4°C for up to 2–3 weeks.

## Step-by-step method details

### Spheroid generation

**Timing: 4 days**

The following steps outline the generation of 96 uniformly sized A549 cancer spheroids.

***Note:*** Additional spheroids may be prepared as a backup.

1. Remove the flask with cultured A549 cells from the incubator and place it inside a laminar flow hood.
2. Aspirate spent medium and wash the flask with 10 mL of sterile PBS.
3. Add 2 mL of warm trypsin-EDTA, ensuring the flask bottom is fully covered.
4. Return the flask to the incubator; incubate for up to 3 min.
5. Bring the flask back to the laminar flow hood.
  a. Add 6 mL of pre-warmed complete cell and spheroid growth culture medium.
  b. With a 10 mL serological pipette, gently mix the cell suspension.
  c. Transfer the cell suspension to a 50 mL sterile conical tube.

***Note:*** The culture medium volume can be adjusted according to cell confluency. An ideal volume is the one that will give a suspension with ≈ 0.5–1.0 × 10^6^ cells/mL.

6. Count and assess A549 cell viability via Trypan Blue exclusion assay in an automated cell counter.

***Alternatives:*** Cell counting can be performed with a classic hemocytometer.

7. Prepare a 10^5^ cells/mL suspension and transfer it to a sterile reagent reservoir.
8. Dispense 100 μL of pre-warmed fresh culture medium into each well of the 96-well ULA-microplate with an 8- or 12-channel pipette.
9. Dispense 100 μL aliquots of the cell suspension into each well of the 96-well ULA-microplate with an 8- or 12-channel pipette.

***Note:*** Combining 100 μL of culture medium with 100 μL of cell suspension will result in seeding 10^4^ cells in 200 μL media/well.

**CRITICAL:** Perform these steps as quickly as possible to prevent cells from settling at the bottom of the reagent reservoir. Mix the suspension in the reagent reservoir with a 25 mL or 50 mL serological pipette before dispensing.

10. Centrifuge the ULA microplate at 500 × *g* for 5 min at 20°C–25°C with no brake. See materials and equipment.
11. Place the ULA microplate in a standard tissue culture incubator and allow spheroids to grow for 4 days.

### Spheroid imaging for automated morphometric analysis

**Timing: 15 min per ULA microplate**

The following steps outline the procedure for imaging individual spheroids after 4 days in culture.

12. Bring the ULA microplate to a brightfield microscope, preferably equipped with a manual (or automated) x/y stage for multi-well plate imaging. This allows stable positioning of the microplate and precise navigation among wells.
13. With the lid in place, systematically capture one image per spheroid/well with a 4× objective. Save all images in one folder.

***Note:*** Refer to Quantification and Statistical analysis for image analysis.

***Note:*** We recommend naming the image files as a number sequence to facilitate subsequent analysis.

### Spheroid migration chamber setup

**Timing: 10 min (for step 14 - Migration chamber setup and loading with 12 spheroids)**

**Timing: 30 min (for step 15 - Migration chamber setup and loading with 96 spheroids)**

The following steps outline the setup of individual migration chambers using 24-well or 6-well TC inserts, loaded with 12 or 96 spheroids, respectively. This setup can be adjusted according to downstream analyses using the parameters provided in Table 1 as a guideline.

***Note:*** Values are applied to each independent experiment run in A549 cancer cells and can be adjusted based on experimental requirements. A larger number of spheroids can be loaded in both 24-well and 6-well TC inserts, as long as overcrowding is avoided.

14. Migration chamber setup and loading with 12 spheroids.
  ***Note:*** If the experimental plan is to perform both morphometric and gene expression analyses, run the migration assay in two (or more) parallel inserts, as each insert supports only one procedure at a time.
  a. In a laminar flow hood, remove the TC insert from its packaging.
  b. Hold the insert firmly with sterile forceps on top of a clean well/dish, while transferring the spheroids from the ULA microplate into the TC insert with a 1000 μL pipette fitted with a cut tip.
    ***Note:*** This procedure allows to collect spent spheroid culture medium percolating through the PET membrane during loading; dispose of it once all spheroids are loaded.
    **CRITICAL:** Avoid overfilling the TC insert with spent medium; see troubleshooting 1 to prevent this.
    **CRITICAL:** Once all spheroids are loaded into the TC insert, check that no spheroid stacking, overlapping, and/or margination have occurred. See troubleshooting 2.
  c. Adjust the final volume of the insert to ≈ 80–100 μL.
  d. Transfer the insert to a well of a 24-well plate.
  e. Fill the bottom well with ≈ 700 μL of medium containing 10% FBS.
    **CRITICAL:** Ensure that no air bubbles are introduced into the insert or the bottom well. See troubleshooting 3.
  f. Cover the 24-well plate with its lid. The migration chamber is now set up.
15. Migration chamber setup and loading with 96 spheroids.
  The following steps outline the setup of a migration chamber using a 6-well TC insert loaded with 96 spheroids (Figure 1).
  a. In a laminar flow hood, place the TC insert on top of an empty, sterile 50 mL conical tube (Figure 1A).
    ***Note:*** Using a 50 mL conical tube allows collection of the spent medium percolating through the PET membrane during spheroid loading. This can be disposed of once the loading procedure is finalized.
  b. Cut a 1000 μL pipette tip with sterile scissors (Figure 1B).
  c. Gently load the pipette tip with ≈10–12 individual spheroids (Figure 1C).
    ***Note:*** It is recommended to load spheroids in batches of ≈ 10–12 to speed up the process and avoid exposing them to conditions outside the controlled environment of the incubator.
  d. Gently release the loaded spheroids onto the TC insert without puncturing the PET membrane (Figure 1D).
  e. Repeat steps c-d until all spheroids are loaded.
    **CRITICAL:** Once all spheroids are loaded into the insert, check that no spheroid stacking, overlapping and/or margination have occurred. See troubleshooting 2.
  f. Adjust the medium volume of the insert to ≈1 mL, avoiding to disturb the spheroids.
  g. Transfer the insert to a well of a 6-well plate with sterile forceps (Figure 1E).
  h. Fill the bottom well with ≈ 3 mL of medium containing 10% FBS.
    **CRITICAL:** Ensure that no air bubbles are introduced into the insert or the bottom well. See troubleshooting 3.
  i. Cover the 6-well plate with its lid. The migration chamber is now set up (Figures 1F and 1G).

**Table 1:** Number of spheroids and TC insert formats according to downstream application

Application	TC insert format	Spheroids per insert	Number of TC inserts	Total number of spheroids
Morphometric image analysis	24-well plate	12	1	12
RT-qPCR	24-well plate	12	4	48
Flow cytometry	6-well plate	96	1	96

**Figure 1:**
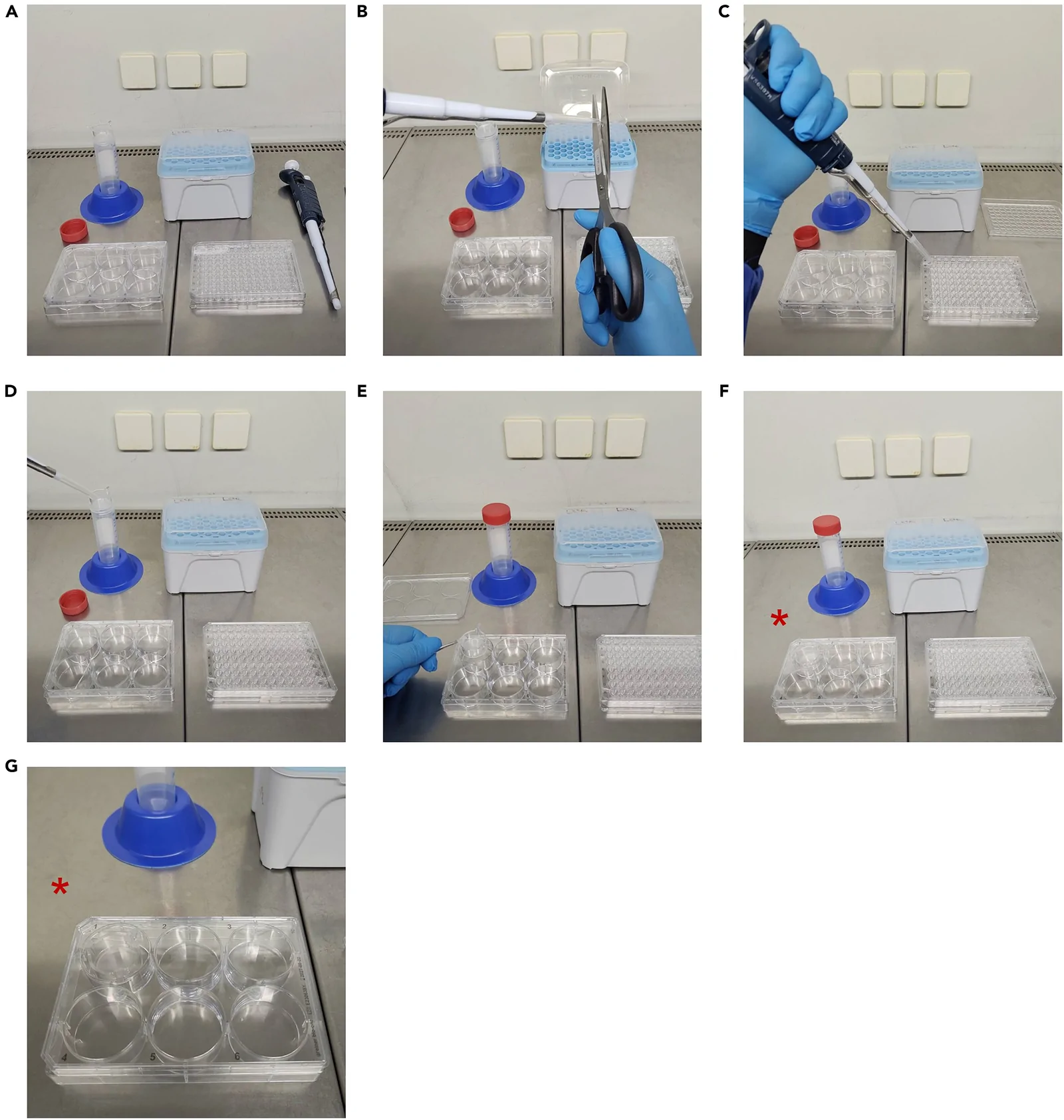
Procedure for loading a 6-well format TC insert with 96 spheroids (A) Place a sterile, 6-well format TC insert on top of a sterile 50 mL conical tube. (B) Cut the end of a 1000 μL pipette tip with sterile scissors. (C) Gently aspirate spheroids from each well of the 96-well ULA microplate. (D) Gently pipette spheroids onto the top surface of the TC insert. Ensure spheroids do not stick to the pipette tip. (E) Once all spheroids are transferred, use sterile forceps to remove the TC insert from the conical tube and place it in a well of a 6-well plate. (F) Cover the 6-well plate with its lid. (G) Close-up view of the mock 6-well plate loaded with a TC insert. Images show a mock procedure; therefore, the depicted materials do not contain spheroids and/or complete cell and spheroid growth medium. The red asterisk in (F) and (G) refers to a mock, loaded 6-well plate.

### Spheroid migration assay

**Timing: 64 h**

This section outlines the steps for carrying out the spheroid migration assay with TC inserts of 24-well and 6-well formats.

16. Transfer the migration chamber to the incubator under standard tissue culture conditions.

***Alternatives:*** The spheroid intrinsic O_2_ gradients can be modified by changing environmental O_2_ via incubation in a hypoxic workstation or chamber.

17. Allow the spheroids to migrate undisturbed for 64 h.

***Note:*** The 64-h migration endpoint can be adjusted based on experimental design and downstream application. Preliminary time-course experiments can be performed to determine the optimal endpoint.

***Note:*** At the migration endpoint, proceed according to the following downstream applications: For 24-well TC inserts, perform morphometric analysis (steps 18–21 plus step 22) or gene expression profiling (steps 21, 23 and 24). For 6-well TC inserts, proceed directly to intracellular hypoxia detection (step 25), where pimonidazole is added to the culture at the migration endpoint before any sample collection or fixation.

### Image acquisition and stitching of the non-migrated spheroid fraction

**Timing: 10 min**

This section describes steps for acquiring and stitching images of the non-migrated spheroid fraction from a single 24-well TC insert. These images are utilized for subsequent morphometric analysis.

18. Remove the 24-well plate from the incubator and bring it to a brightfield microscope.

***Note:*** Inspect the insert and ensure that no air bubbles are present following handling of the multi-well plate. See troubleshooting 3.

19. With a 2× objective, capture and save four consecutive images covering the whole PET membrane of the insert.

**CRITICAL:** Ensure adjacent microphotographs share ≈ 10%–20% overlap; this allows the stitching plugin^6^ to render a final image covering the whole PET membrane. See troubleshooting 4 if stitched images are blurred and/or contain duplicated features in the final tile.

**Pause point:** Image stitching can be performed at a later stage.

20. To stitch the images:
  a. Open Fiji.
  b. Go to Plugins → Stitching → Grid/Collection stitching → Unknown position. Leave ‘Order - All files in directory’ tab as it is by default.
    ***Alternatives:*** Plugins → Stitching → Grid/Collection stitching → Sequential Images.
  c. Click ‘OK’.
  d. In the ‘Directory’, click ‘Browse’ to localize the file with the saved images.
  e. Click ‘Open’.
  f. Use default parameters as shown in the dialog box and click ‘OK’.
  g. A new dialog box will open to ‘Confirm files’. Check that all the images that need to be stitched are shown and checked.
  h. Click ‘OK’.
  i. A ‘Fused’ 8-bit image is now generated.
  j. Save it as JPEG.
  k. Crop stitched images with Fiji, excluding the TC insert side wall. Save cropped images.
    ***Note:*** These images are used for morphometric analysis together with the migrated-fraction images acquired at the end of step 22 (see quantification and statistical analysis).

### Collection of the non-migrated spheroid fraction

**Timing: 10 min or 20 min (for 24-well or 6-well TC insert)**

This section describes the steps for collecting the non-migrated (top) spheroid fraction following the migration assay in 24-well or 6-well TC inserts.

***Note:*** Volumes collected in steps 21d and 21f can be discarded when proceeding to crystal-violet staining for morphometric analysis; in this case, omit steps 21i-l and continue to step 22.

***Note:*** These collection steps are also used to recover the pimonidazole-incubated non-migrated fraction when called from step 25d.

21. Sample collection from one 24-well or one 6-well TC insert.
  ***Note:*** In the following steps, the first volume refers to a 24-well TC insert and the second to a 6-well TC insert.
  a. Remove the 24-well or 6-well TC insert from its well.
  b. Add ≈ 200 μL or ≈ 2 mL of PBS to the TC insert.
  c. With a nonwoven swab, gently lift/detach the spheroids inside the insert.
    ***Note:*** A degree of spheroid disaggregation is expected at this stage due to gentle lifting/detaching.
    **CRITICAL:** See materials and equipment for the relevance of using a nonwoven swab at this step.
  d. Collect this volume in a 15 mL conical tube with a 1000 μL pipette fitted with a cut tip.
    ***Note:*** Low-retention pipette tips are recommended at this stage.
  e. Add ≈ 200 μL or ≈ 2 mL of PBS to the insert and gently lift any remaining spheroids using the same nonwoven swab.
  f. Collect this volume in the same 15 mL conical tube.
    ***Note:*** Steps e-f can be repeated if any non-migrated spheroid fraction is suspected to remain in the insert.
    ***Alternatives:*** If the total collection volume exceeds 15 mL, a 50 mL conical tube is recommended.
  g. Gently wash the inside of the insert with ≈ 500 μL or ≈ 2 mL of PBS using a cotton swab, applying circular movements.
  h. Discard the PBS.
    **CRITICAL:** Never apply pressure to the PET membrane or puncture it, as it can be easily damaged and the samples lost.
    **CRITICAL:** Do not disturb the migrated fraction on the underside of the PET membrane during these steps.
    **CRITICAL:** Keep the insert, now containing only the migrated fraction, for either crystal violet staining or collection for downstream analyses.
  i. Centrifuge sample at 500 × *g* for 5 min at 20°C–25°C.
  j. Aspirate supernatant.
  k. Wash the pellet once with ≈ 2 mL or ≈ 4 mL of PBS.
  l. Centrifuge the sample again; keep the pellet.
    **Pause point:** The pellet can be flash-frozen and stored at −80°C if not used immediately for gene expression profiling.

### Staining and imaging or collection of the migrated-spheroid fraction

**Timing: 40 min active time + 48 h insert drying (for step 22 - Staining and imaging of the migrated-spheroid fraction (bottom))**

**Timing: 30 min or 40 min (for step 23 - Collection of the migrated-spheroid fraction (bottom fraction))**

This section describes the steps for staining and imaging the migrated-spheroid fraction and for collecting a separate migrated-fraction sample for downstream analyses.

**CRITICAL:** Separate sample sets are required if staining/imaging and collection for downstream analyses are performed in parallel. Samples used for staining and imaging are not suitable for subsequent collection.

***Note:*** At this point in the protocol, the non-migrated (top) fraction has already been collected in step 21; thus, the TC insert contains only the migrated (bottom) fraction.

22. Staining and imaging of the migrated-spheroid fraction (bottom).
  The following steps describe crystal-violet staining and imaging of the migrated spheroid fraction, optimized for use with 24-well TC inserts.
  ***Note:*** The following steps can be executed on a lab benchtop and refer to one 24-well TC insert.
  a. Add ≈ 200 μL of PBS to the insert and gently wash it with a cotton swab, applying circular movements.
  b. Aspirate and discard all contents from the insert.
    **CRITICAL:** Never apply pressure to the PET membrane or puncture it, as damage will interfere with image analysis and/or cause sample loss.
  c. Transfer the TC insert into a clean well of a 24-well plate prefilled with ≈ 700 μL of crystal violet solution.
    **Table:** Crystal violet staining solution

Reagent	Final concentration	Amount
Crystal violet	0.5% w/v	0.25 g
Methanol	20% v/v	10 mL
ddH_2_O	–	Fill up to 50 mL
Total	–	50 mL
    ***Note:*** Prepare the amount of solution needed before use and discard any remaining afterwards.
    ***Alternatives:*** Commercially available, ready-to-use crystal violet solutions.
  d. Stain the insert for ≈ 10 min at 20°C–25°C.
  e. Wash the insert as follows:
    i. Remove the insert from the well.
    ii. Gently aspirate the crystal violet solution with a pipette.
    iii. Add ≈ 500 μL of PBS to the insert and gently clean it with a cotton swab, applying circular movements.
    iv. Discard PBS and repeat step iii as needed until the insert is clean.
      **CRITICAL:** Ensure no cotton fibers remain on the PET membrane, as these will interfere with image analysis.
  f. Allow the insert to air dry for 48 h in the dark at 20°C–25°C.
  g. Place the stained insert on a clean glass slide positioned at the center of the microscope.
  h. Use a 4× objective to capture nine consecutive images in a 3-by-3 grid.
    **CRITICAL:** Ensure adjacent microphotographs share ≈ 10%–20% overlap; this allows the stitching plugin^6^ to render a final image covering the whole PET membrane. See troubleshooting 4 if stitched images are blurred and/or contain duplicated features in the final tile.
  i. Save and stitch the images as outlined in step 20.
    ***Note:*** Image analysis (see quantification and statistical analysis) can be performed at the end of this step or at a later time.
    ***Note:*** Store the stained insert in the dark at 20°C–25°C in a dust-free, clean box.
23. Collection of the migrated-spheroid fraction (bottom fraction).
  The following steps outline the procedure for collecting the migrated-spheroid fraction from one 24-well or 6-well TC insert.
  ***Note:*** These collection steps are also used to recover the pimonidazole-incubated migrated fraction when called from step 25d.
  ***Note:*** In the following steps, the first volume refers to a 24-well TC insert and the second to a 6-well TC insert.
  a. Transfer the 24-well or 6-well TC insert containing the migrated fraction to a clean well prefilled with ≈ 700 μL or ≈ 2 mL of pre-warmed (37°C) trypsin.
  b. Transfer the 24-well plate or 6-well plate to a shaker.
  c. Gently shake for 10 min at 37°C.
    ***Alternatives:*** This timing can be adjusted based on when cells detach and become visibly suspended in the well.
  d. Lift the insert and inspect the well for a visible cell suspension. See troubleshooting 5 for incomplete cell detachment in this step.
    ***Note:*** If multiple inserts are used in the same independent experiment (see Table 1), use the same trypsin and well to detach and collect additional migrated fractions.
  e. Collect the cell suspension into a 15 mL tube.
  f. Add 2 mL or 4 mL of complete cell and spheroid growth medium.
  g. Pipette and centrifuge the sample at 500 × *g* for 5 min at 20°C–25°C.
  h. Aspirate supernatant.
  i. Wash the pellet once with ≈ 2 mL or ≈ 4 mL of PBS.
  j. Centrifuge the sample again; keep the pellet.
    **Pause point:** The pellet can be flash-frozen and stored at −80°C if not used immediately for gene expression profiling.

### Gene expression profiling of the non-migrated and migrated spheroid fractions

**Timing: 3 h**

This section describes the steps for processing collected non-migrated and migrated cancer cell populations for gene expression analysis, enabling paired comparison of their transcriptional profiles.

***Note:*** The number of spheroids and TC inserts used per independent biological experiment is specified in Table 1.

24. Processing of pellets collected at the end of steps 21 and 23.
  a. Extract RNA from each sample following the manufacturer’s instructions.
  b. Measure RNA quality and concentration with a spectrophotometer.
  c. Proceed with cDNA synthesis.
  d. Proceed with RT-qPCR.

***Alternatives:*** We use our validated and routinely used reagents and instruments (see key resources table) to carry out steps 24a-d. However, equivalent commercially available reagents and instruments can be used in these steps, according to each laboratory's established routine, provided that the manufacturer's recommended conditions are followed and that the same reagents and conditions are applied consistently to the paired migrated and non-migrated fractions within experiments.

***Note:*** Examples of gene expression data analysis can be found in Schito & Rey-Keim.^1^

### Detection of intracellular hypoxia in non-migrated and migrated spheroid fractions

**Timing:** ≈ **3 h active time +** ≈ **68 h incubation time**

This section describes the steps for adding pimonidazole to the migration chamber, enabling paired detection of intracellular hypoxia in non-migratory and migratory populations.

***Note:*** The following procedure is performed using a 6-well TC insert.

***Note:*** Unless otherwise specified, centrifugation steps in this section are performed at 500 × *g* for 5 min at 20°C–25°C.

25. At the end of the migration assay, bring the 6-well plate with the migration chamber to a laminar flow hood.
  a. Measure the volume of spent medium in the bottom well and add pimonidazole solution to achieve a final concentration of 200 μM.
    ***Note:*** A pimonidazole stock concentration of 20 mM in sterile PBS will result in adding 30 μL to a remaining spent medium volume of 3 mL.
  b. Gently swirl the 6-well plate to ensure even pimonidazole distribution.
  c. Incubate the 6-well plate in the tissue culture incubator for 3 h.
  d. Collect the pimonidazole-incubated non-migrated fraction following the procedure described in step 21, and the pimonidazole-incubated migrated fraction following the procedure described in step 23. Each fraction yields a separate pellet.
  e. Non-migrated fraction only: trypsinize the pellet as follows:
    i. Add 300 μL of pre-warmed (37°C) trypsin to the pellet; pipette to favor cell-cell dissociation.
    ii. Incubate for ≈ 5–10 min at 37°C under gentle rotation.
    iii. Pipette to further favor cell-cell dissociation.
    iv. Add 2 mL of complete cell and spheroid growth medium; pipette gently.
    v. Centrifuge and discard the supernatant.
    vi. Wash the pellet with 1000 μL of PBS.
    vii. Centrifuge and discard the supernatant.
      ***Note:*** This dissociation is required only for the non-migrated fraction, which is collected as a pellet of spheroids and spheroid fragments following step 21. The migrated fraction is recovered already as a dissociated cell pellet following step 23.
  f. Both fractions: from this point, the non-migrated and migrated fractions are processed identically. Resuspend and fix each pellet with a final 2% paraformaldehyde in PBS for 48 h at 4°C.
  g. Centrifuge samples and discard the supernatant.
  h. Wash each pellet with 1000 μL of 1% BSA in PBS.
  i. Centrifuge samples and discard the supernatant.
  j. Resuspend each pellet with 1000 μL of primary antibody staining solution.
    ***Note:*** Pool 10–20 μL from each sample fraction in 500 μL of staining solution without primary antibody, and process this sample in parallel with the others. This will serve as the fluorescence-minus-one control.
    **Table:** Primary antibody staining solution

Reagent	Final concentration	Amount
BSA	1% (w/v)	30 mg
Triton X-100	0.05%	1.5 μL
Anti-pimonidazole antibody	1:250	12 μL
PBS	–	Up to 3 mL
Total	–	3 mL
    ***Note:*** Prepare fresh before use and discard any remaining solution afterwards. Volumes in the table already include an excess to account for dead volume.
  k. Incubate samples for 16 h at 4°C under gentle rotation.
  l. Centrifuge samples and discard the supernatant.
  m. Wash each pellet with 1000 μL of 1% BSA in PBS.
  n. Centrifuge samples and discard the supernatant.
  o. Resuspend each pellet with 1000 μL of secondary antibody staining solution.
    **Table:** Secondary antibody staining solution

Reagent	Final concentration	Amount
BSA	1% (w/v)	40 mg
Alexa Fluor-647 conjugated secondary antibody	1:1000	4 μL
PBS	–	Up to 4 mL
Total	–	4 mL
    ***Note:*** Prepare fresh before use and discard any remaining solution afterwards. Volumes in the table already include an excess to account for dead volume.
  p. Incubate for 1 h in the dark at 20°C–25°C under gentle rotation.
  q. Centrifuge samples and discard the supernatant.
  r. Wash each pellet with 1000 μL of 1% BSA in PBS.
  s. Centrifuge samples and discard the supernatant.
  t. Resuspend each pellet with 1000 μL of 1% BSA in PBS.
  u. Analyze samples with a flow cytometer equipped with a 638 nm laser.
    ***Alternatives:*** A range of primary anti-pimonidazole antibodies and compatible secondary conjugated antibodies are commercially available for use with these steps.
    ***Note:*** We run our samples in a CytoFLEX S and perform data analysis with CytExpert and/or FlowJo.
    ***Note:*** We add pimonidazole at the end of the 64-h migration period for 3 h, while the spheroids are viable and metabolically active, forming irreversible adducts in cells that are hypoxic during this 3-h labeling time. Only after this incubation are both migrated and non-migrated cell fractions collected, fixed, and processed for antibody-based detection of intracellular hypoxia by flow cytometry. Because adducts are retained within hypoxic cells, the pimonidazole-positive status remains even in the face of subsequent reoxygenation. Therefore, pimonidazole labeling reflects hypoxic status during this defined endpoint window rather than the entire migratory trajectory; in this setup, hypoxia is determined experimentally in each fraction and is not inferred from spheroid geometry (i.e., core versus rim), as factors such as oxygen consumption and local cell density impose non-linear hindrances upon inference approaches. Moreover, since hypoxia can be transient or intermittent, the endpoint design of this protocol aims at an integrated hypoxic signal rather than short-term O_2_ variations. Users requiring resolution of these fluctuations in O_2_ dynamics may adapt the protocol to include HRE-based fluorescent reporters, GFP-ODD or related fusion reporters for live imaging, as discussed in Schito & Rey-Keim.^1^

## Expected outcomes

Successful implementation of the HyPIC-3D protocol is expected to yield two distinct sample fractions containing migrated and non-migrated cancer cell subpopulations, respectively, derived from the same pool of spheroids. These fractions are amenable to morphometric analysis and subsequent molecular and cellular profiling using applications such as RT-qPCR for gene expression analysis and flow cytometry for quantification of intracellular hypoxia; due to its versatility, this protocol can also be applied to single-cell and bulk omics approaches.^1^ The resulting datasets can be analyzed in a paired, side-by-side comparative framework, with each pair serving as its own control, thereby reducing intrinsic sample variability and increasing statistical power; this approach offers insights into the molecular mechanisms driving migratory heterogeneity between the two populations.^1^ Similarly, application of HyPIC-3D across multiple cancer types is expected to uncover both distinct and conserved regulators of migration, providing mechanistic insights into the early metastatic behaviors that define malignant tumors.^1^

## Quantification and statistical analysis

Morphometric image analysis within HyPIC-3D is carried out using individual spheroid images, and unstained versus crystal violet-stained TC insert images by four separate automated or semi-automated scripts run in Fiji (https://fiji.sc/), an open-source software implementation of ImageJ. Fiji is available for Windows, macOS and Linux operating systems, and should be pre-installed in the computer used for analysis. After opening Fiji, go to Plugins → Macros → Install and select the downloaded script, which will become available under the menu Plugins → Macros.

***Note:*** It is important to install and run one script at a time, and it is suggested to organize input images within single folders for analysis.

***Note:*** For simplicity, all macros provide areas and linear measurements in pixels.

**CRITICAL:** Image calibration should be ensured prior to acquisition by using in-built spatial calibration or a micro-photograph of a graduated microscope slide allowing to convert pixels to micrometers. This is necessary for standardization of morphometric data, ensuring that areas and linear measurements are expressed in μm^2^ and μm, respectively.

### Automated morphometric image analysis for individual spheroids

Script 00 performs batch conversion of RGB to required 8-bit grayscale input images, whereas Script 01 extracts morphometric parameters (i.e., cross-sectional area, perimeter, circularity and Feret’s caliper diameters) on a per-image basis, assuming that each image contains a single spheroid, before transfer onto a TC insert. Additionally, a binary image showing the cross-sectional area of each input image is included for validation. Dust particles and noise cleanup thresholds can be set manually based on minimum area (default: >5 × 10^4^ μm^2^) and circularity (default: >0.1). The resulting text file is saved automatically in the indicated output folder, and the data can be copied, pasted or imported into spreadsheet software for analysis.

***Note:*** Parameters can be left as default or adjusted as necessary.

### Semi-automated image analysis of migratory phenotypes

In order to quantify the migration patterns of 3D spheroids as they traverse the TC insert membrane, images should contain the whole TC insert area within a single image. Migration ratios (R_m_)^1^ are calculated based on cross-sectional and migrated areas that are expressed in consistent absolute units (i.e., μm^2^ or mm^2^).

At endpoint, cross-sectional areas of non-migrated spheroids laying on the top of the membrane are measured with Script 02 (output header: ‘Total Area’), whereas the migrated area and optical density (OD) bands after crystal violet-staining are measured with Script 03. Combining these two script outputs enables the calculation of R_m_, which reflects the pattern and extent of migration, and provides data on the OD of the crystal violet-stained inserts,^1^ used to determine the post-migration density distribution quantifying cell overlap/stacking. Accordingly, Script 03 pragmatically quantifies OD by binning integrated ODs into low (0.202–0.472), middle (0.472–0.734) and high (0.734–2.407) bands whilst measuring the total area of migrated cells constrained within said OD bands (in μm^2^).

Different microscopes can be used for image acquisition as long as calibration information is recorded prior to taking microphotographs. Alternatively, scaling calibration in stitched images can be derived by measuring the TC insert diameter in pixels within Fiji and using this measurement to calculate a scaling factor using the TC insert area as stated in the manufacturer’s specifications. For example, if a 24-well TC insert has a growth area of 0.3 cm^2^ (equal to 3×10^7^ μm^2^), insert diameter will be $2\times \sqrt{\frac{3\times 10^{7}}{\pi }}$ or ≈6,180 μm. This value can then be used to perform spatial calibration in Fiji by drawing a line across the diameter of the TC insert in the stitched image and using the pixel value as well as the calculated diameter to obtain the spatial calibration factor expressed as pixels per 1,000 μm as required in all scripts. This calculation can then be used to replace calibration grid microphotographs as a prerequisite for Script 02 and Script 03. Notwithstanding, the calibration microphotograph method should be preferred for simplicity.

**CRITICAL:** Ensure that constant illumination is maintained across sessions in order to obtain accurate OD data.

### Statistical analysis of paired samples

Migration areas and ratios behave empirically as log-normal distributions, therefore making non-parametric, paired statistical analyses preferable (e.g., Wilcoxon’s paired signed-rank tests) as included in standard statistical software (e.g., GraphPad Prism). If non-parametric tests are unavailable, logarithm-transformed data can be used for parametrization and analysis with paired Student’s t-tests or repeated-measures ANOVA.

## Limitations

The applicability of the HyPIC-3D protocol is linked to the ability of the cancer cell line under investigation to produce spheroids cohesive enough to withstand the transfer step between culture vessels. Consequently, spheroid generation conditions should be optimized before starting this protocol, with attention to methods influencing spheroid aggregation and self-assembly, compaction, initial seeding density and growth conditions. It is also worth noting that the use of exogenous matrices to enhance spheroid formation was not assessed in HyPIC-3D.^1^ Another factor to consider is how readily the chosen cell line migrates in 3D *in vitro* microenvironments, which influences sample yield. Low migratory capacity may result in limited numbers of migrated cells, thereby restricting downstream applications. Characterizing baseline 3D motility and chemotactic response in pilot experiments is advisable.

## Troubleshooting

### Problem 1

Spheroid loading leading to TC insert volume overfilling. Related to step 14.

### Potential solution

This can be avoided by implementing the following procedure.

• Transfer 1–3 spheroids with their spent medium (≈100–150 μL) into the insert slowly, allowing the medium to percolate through the PET membrane by gravity.
• Wait for the spheroids to settle at the bottom of the insert (this occurs within a few seconds).
• Load additional spheroids once a thin layer of medium is left covering the membrane.
• Repeat these steps until all spheroids are loaded.

### Problem 2

Stacking, overlapping, and/or margination of spheroids when loading TC inserts. Related to steps 14–15.

### Potential solution

• Dispense spheroids carefully onto the inserts to avoid stacking, overlapping, or margination, which can interfere with image analysis.
• If stacking, overlapping, or margination still occur, gently tap the inserts to disperse the spheroids. Inspect the inserts under a microscope with the plate lid on.

### Problem 3

Air bubbles trapped within the TC insert and/or between the insert and the bottom well. Related to steps 14, 15 and 18.

### Potential solution

• Gently tap the TC insert and/or the 24-well or 6-well plate and inspect under a microscope.
• If air bubbles persist, remove them by carefully aspirating or disrupting them with a pipette or pipette tip (1–10 μL), or by gently moving the insert to release them.

**CRITICAL:** Avoid disturbing and/or displacing the spheroids.

### Problem 4

Blurred stitched images and/or duplicated features in the final tile. Related to steps 19 and 22.

### Potential solution

• Ensure that shared image borders fall within ≈ 10%–20% of width/height for any given pair of adjacent microphotographs. Overlap below this range might prevent the plugin from registering adjacent tiles; overlap above it might generate large redundant areas that increase the risk of incorrect tile placement and duplication.
• Remove duplicate or near-identical images.
• Maintain a consistent grid pattern covering the entire TC insert (e.g., left-to-right and top-to-bottom).
• Re-run the stitching plugin^6^ in Fiji.

### Problem 5

Incomplete detachment of the migrated-fraction from the bottom of the PET membrane during trypsinization. Related to step 23.

### Potential solution

• Ensure that the well contains sufficient trypsin (or an equivalent detachment solution such as Accutase) to fully cover the PET membrane.
• Increase detachment time and/or shaker speed.

## Resource availability

### Lead contact

Further information and requests for resources and reagents should be directed to and will be fulfilled by the lead contact, Luana Schito (luana.schito@ucd.ie).

### Technical contact

Technical questions on executing this protocol should be directed to and will be answered by the technical contact, Luana Schito (luana.schito@ucd.ie).

### Materials availability

This study did not generate new unique reagents.

### Data and code availability

This protocol does not generate new code. The scripts listed in the key resources table and applied to this protocol are those reported in the associated research article.^1^

## Acknowledgments

Research in the authors’ laboratories is supported by the 10.13039/501100001631UCD Ad Astra Fellows Programme, the UCD Seed Funding Scheme (grants R20841, R20842, and SF1916 to L.S. and R20849 and R20850 to S.R.-K.), and the Eric Reid Fund for Methodology from The Biochemical Society (GB) (L.S.). We thank Associate Professor Alfonso Blanco (Flow Cytometry Core, UCD Conway Institute) for expert technical advice and Dr. Tracy Mullen (UCD Conway Institute) for excellent laboratory management assistance.

## Author contributions

Conceptualization L.S. and S.R.-K.; formal analysis L.S. and S.R.-K.; investigation L.S. and S.R.-K.; methodology L.S. and S.R.-K.; software L.S. and S.R.-K.; supervision L.S. and S.R.-K.; validation L.S. and S.R.-K.; visualization L.S. and S.R.-K.; writing – original draft L.S. and S.R.-K.; writing – review and editing L.S. and S.R.-K.; funding acquisition L.S. and S.R.-K.; project administration L.S. and S.R.-K.

## Declaration of interests

The authors declare no competing interests.
